# Diagnostic Performance of Ultrasound in Adult Appendicitis. A Retrospective Review of 331 Cases

**DOI:** 10.1002/jmrs.70079

**Published:** 2026-03-11

**Authors:** Alistair Lock, Martin Necas

**Affiliations:** ^1^ Waikato Hospital Hamilton New Zealand

**Keywords:** abdominal pain, adult, appendectomy, appendicitis

## Abstract

**Introduction:**

Definitive and accurate diagnosis of acute appendicitis is essential to reduce morbidity, yet ultrasound demonstrates variable performance which has generally improved over time with advancing technology and user expertise. This study aimed to evaluate the diagnostic accuracy of ultrasound for appendicitis in adults in our institution and document alternative diagnoses detected.

**Methods:**

We conducted a retrospective review of all abdominal ultrasound examinations performed between January 2021 and December 2021 for patients ≥ 16 years where appendicitis was a clinical query. Reports were categorised as positive or negative based on standardised interpretive phrases. Clinical records, operative findings, histology and follow‐up determined true outcomes. Inconclusive examinations (non‐visualised appendix) were classified as negative for analysis.

**Results:**

The cohort comprised 331 patients (92% female; mean age 28 years). Appendicitis prevalence was 16% (53/331). For the detection of appendicitis, ultrasound demonstrated sensitivity of 60%, specificity 98%, PPV 82% and NPV 93%. Alternative diagnoses (*n* = 108) were more frequent than appendicitis by 2:1 and included haemorrhagic ovarian events, hydrosalpinx, mesenteric adenitis and other gynaecological or abdominal pathologies.

**Conclusions:**

Ultrasound demonstrated modest sensitivity but high specificity for diagnosing appendicitis in adults, consistent with international data when inconclusive cases are included. This shows that in the absence of any ultrasound features, appendicitis is unlikely. Alternative findings were twice as common as appendicitis, highlighting the value of ultrasound as a primary imaging modality for the assessment of patients with right lower quadrant pain.

## Graphical abstract

The sensitivity of ultrasound in the detection of appendicitis had a prevalence of 16% in our predominantly young female cohort, with alternative gynaecological and abdominal diagnoses identified approximately 2:1. The sensitivity for ultrasound to detect appendicitis was 60%, with specificity of 98% and negative predictive value (NPV) of 93%. In the absence of any ultrasound features, appendicitis is unlikely.

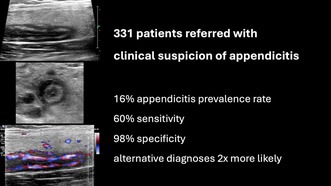

## 1 Introduction

Timely diagnosis of acute appendicitis is required for effective management and reduction of morbidity. However, despite improvements in ultrasound technology, acute appendicitis remains a considerable diagnostic challenge for clinicians, sonographers and radiologists. The published sensitivities and specificities for the detection of appendicitis by ultrasound are wide‐ranging, have changed significantly over time and are difficult to generalise for specific patient populations and ultrasound users [1, 2].

The only New Zealand study on the diagnostic performance of ultrasound in the diagnosis of acute appendicitis conducted in 2015 showed a disappointing sensitivity of ultrasound (around 50%) [3]. The specificity was high at 99%, but this was partly attributable to the low prevalence of appendicitis in the cohort (14%) [3]. Positive predictive value (PPV) and negative predictive value (NPV) were 84% and 93% respectively at this time.

A meta‐analysis from Fu et al. published in 2021 showed average overall sensitivity and specificity values of 77% (range 50%–100%) and 60% (range 0%–97%) for ultrasound diagnosis of acute appendicitis from 18 studies published between 2010 and 2021 [4]. A further systematic review analysing 21 studies dated 1998–2021 showed average overall sensitivity and specificity values of 81% and 87% [5]. The two meta‐analyses included mixed cohorts with paediatric as well as adult patients.

More recent studies have shown better diagnostic performance perhaps reflecting advancing technology with high‐density ultra‐broadband transducers, increased system sensitivity and improvements in beamforming and signal processing, as well as evolving sonographer skill/training. A Spanish study from 2022 showed a very high positive predictive value of 97% in 278 patients [6]. Another study from Switzerland in 2022 showed sensitivity, specificity, PPV and NPV values of 90%, 94%, 88% and 95% respectively in a larger patient group of 508 patients across 2 years [7]. However, the sensitivity and specificity of different studies are dependent on pre‐test probability of appendicitis, experience of the imaging team, as well as the form of analysis. In particular, some studies only include patients where the appendix was positively visualised and exclude up to 40% of indeterminate cases from analysis [7].

In combination with newer technology, greater user awareness and improved examination techniques, the diagnostic performance of ultrasound should also be improving. The aim of our study was to determine the specificity, sensitivity, positive and negative predictive values for ultrasound in diagnosing acute appendicitis in adults. The secondary aim was to document alternative diagnoses revealed through ultrasound imaging in our cohort.

## 2 Methods

This study was approved by the local institutional ethics committee, Health New Zealand/Te Whatu Ora Waikato Clinical Audit Support Unit. Individual patient consent was not required, as the study involved retrospective analysis of existing imaging and report data.

This study was a retrospective sequential review of all abdominal ultrasound examinations performed over the course of 1 calendar year, between 1 January 2021 and 31 December 2021, on adult patients (16 years or older) where ‘appendicitis’ was listed as the clinical question (or one of the clinical questions) on the referral form.

Patients were identified using a search of the radiology information system (Karsima, Kestral, Auckland). Their ultrasound reports were reviewed and categorised into scan‐positive and scan‐negative groups for the presence of appendicitis. The scan result was defined as positive when the report included affirmatory phrases such as: ‘correlates with appendicitis’, ‘consistent with appendicitis’, ‘suggestive of appendicitis’, ‘probable appendicitis’, ‘suspicious for appendicitis’. The scan was defined as negative when no convincing features of appendicitis were reported and the report contained negative or neutral phrases such as: ‘no evidence of appendicitis’, ‘no indirect signs of appendicitis’, ‘appendicitis not visualised’, ‘appendicitis cannot be ruled out’. The decision to place a patient into the positive or negative categories was decided by individual authors based on the above definitions, with select cases jointly reviewed if the wording was deemed indeterminate. Patients were included in analysis whether the appendix was positively identified or not.

True positive and true negative cases of appendicitis were determined by review of clinical records including follow‐up imaging, surgical reports, histology and discharge summaries. The gold standard for the diagnosis of appendicitis was a positive histology report. The true negative group of patients included those who (a) discharged following a negative ultrasound scan and did not re‐present (b) proceeded with advanced imaging such as CT or MRI scan and an alternative pathology found (c) proceeded with an exploratory laparoscopy but without appendicectomy, (d) underwent appendicectomy and histology report showed negative results for appendicitis.

All examinations were performed by qualified sonographers with a minimum of a postgraduate degree qualification. The examination images were then reviewed and reports authorised by staff radiologists. The examination protocol for the assessment of RIF pain includes: appendix, terminal ileum, caecum, urinary bladder, right kidney, gallbladder and in female patients pouch of Douglas, right ovary and right adnexa. In the presence of a gynaecological abnormality, the examination is extended to include transvaginal ultrasound. All examinations were booked at standard booking times of 30 min including reporting, and were performed using Philips Epiq Elite systems with the latest versions of software available at the time of the examinations.

## 3 Results

**FIGURE 1:**
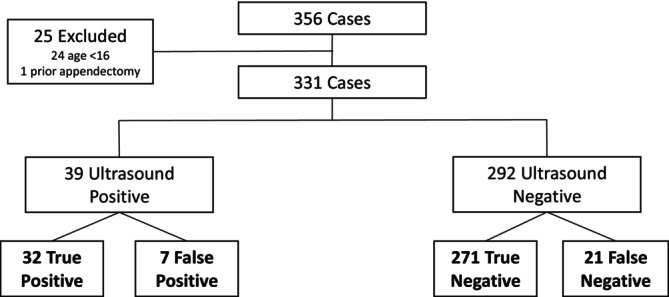
Exclusion criteria and outcomes for included cases.

**FIGURE 2:**
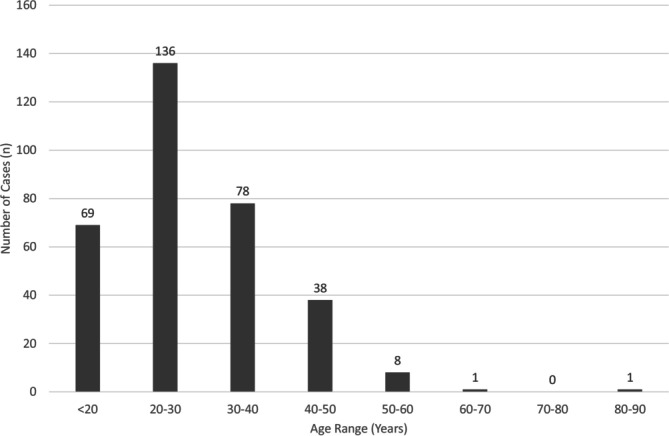
Patient age distribution.

The patient cohort comprised 331 patients (92% females and 8% males) with an age range of 16–80 years (mean = 28, median = 25). Figure 1 demonstrates patient selection and outcomes. Age category histogram is shown in Figure 2.

The most common indications for examination were a combination of: right lower quadrant (RLQ) pain (89%), raised inflammatory markers (38%), nausea (33%), vomiting (19%) and pyrexia (15%). The majority of patients were referred from inpatient ward services (76%) with others from the emergency department (23%) and a small number directly from general practitioners (1%).

There were 53 cases of appendicitis, giving a prevalence rate of 16%. The oldest patient with acute appendicitis was 49 years old. The sensitivity, specificity, positive and negative predictive value of ultrasound in the diagnosis of appendicitis was 60%, 98%, 82% and 93% respectively.

**FIGURE 3:**
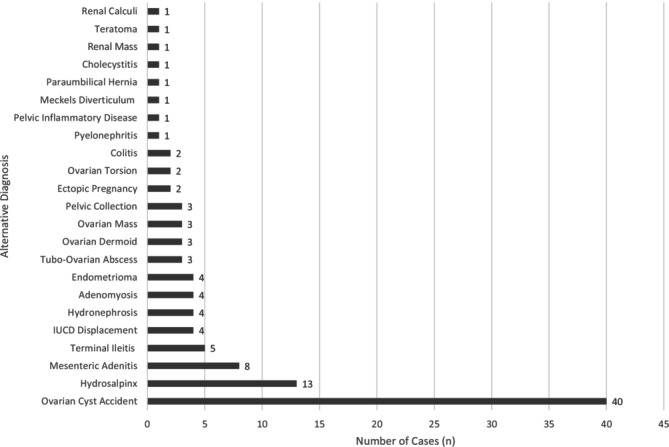
Alternative diagnoses made on ultrasound.

A large number (*n* = 108) of other findings were uncovered in our patient cohort (Figure 3), including haemorrhagic ovarian event (*n* = 40), hydrosalpinx (*n* = 13), mesenteric adenitis (*n* = 8), terminal ileitis (*n* = 5), endometrioma (*n* = 4), adenomyosis (*n* = 4), hydronephrosis (*n* = 4), IUCD displacement (*n* = 4) and others. The number of alternative findings outnumbered appendicitis approximately 2:1.

## 4 Discussion

The sensitivity for the detection of appendicitis in this study was 60%, versus 50% a decade prior [3], a modest improvement of 10%. Specificity, PPV and NPV were 98%, 82% and 93% in this study and therefore very similar to a decade prior (99%, 84% and 93% respectively). The referral pattern was shown to be stable, with prevalence of appendicitis in patients referred for imaging found to be 16%, compared to 14% a decade ago.

The sensitivity, specificity, PPV and NPVs for detecting appendicitis in our adult population was comparable to other tertiary hospitals in Europe [6, 7]. Some authors report higher sensitivity, but this is largely attributable to different methodology. For instance, Lehmann et al. achieved a sensitivity of 90% but only by removing 200 patients (39.4% of their total cohort) from analysis because these patients had a sonographically ‘inconclusive’ examination. It is important to recognise that some patients with a non‐visualised appendix may in fact harbour acute appendicitis. It would therefore be more appropriate to identify these patients and treat them as ‘false negative’ findings on ultrasound instead. By excluding these patients from analysis, the diagnostic performance of ultrasound will appear greater.

In our study, we treated inconclusive (i.e., appendix not visualised) ultrasound examinations as negative ultrasound examinations; an approach we believe to be more clinically relevant. The false negative patients in our cohort had a negative ultrasound examination and then proceeded for CT (2 patient), MRI (1 patient) or direct to surgery (18 patients), where appendicitis was detected. The appendix was visualised in only 1 of the 21 false negative ultrasound studies from our data, showing that this methodology provides more real‐world diagnostic performance.

Another contributing factor for the discrepancy in sensitivity between our study and other published literature is the low pre‐test probability of appendicitis in our population, with the overall prevalence of appendicitis in our cohort being 16%. This is much lower than other retrospective studies which suggest a prevalence of 25%–30% [4, 5, 6, 7]. In our analysis, we noted that many ultrasound examinations were not targeted to the right iliac fossa but also included renal and gynaecological queries. We believe this is because our referrers were utilising ultrasound as a screening tool to assess for a broader range of differentials of patients with acute abdominal pain, which ultimately reduces the prevalence of appendicitis for our population. Improving in this referral pattern or using a prospective study design would improve the sensitivity.

Moreover, alternative diagnoses revealed through ultrasound outnumbered the cases of true appendicitis by approximately 2:1. The most common alternative diagnoses were gynaecological in nature (Figure 3). This supports the ongoing use of ultrasound as an initial triage tool in patients with RLQ pain.

The alternative imaging modality for the diagnosis of appendicitis is CT; however, we note a female predominance of cases referred for ultrasound (92%) with a median age of 25 years in our study cohort. Given the rapidly growing use of ionising radiation in medicine and the potential carcinogenic risk associated with this [8, 9, 10], it would seem prudent to avoid the use of radiation in diagnosis wherever possible, particularly in the female population of child‐bearing age.

Early diagnosis of appendicitis is essential for preventing complications such as perforation, peritonitis/collections and sepsis which can add significant patient inpatient stay time and healthcare costs [11, 12, 13]. Depending on local resourcing, ultrasound can often be deployed more rapidly than CT where booking can be limited by clinical priorities such as stroke or trauma cases, again favouring ultrasound as the primary imaging modality for the investigation of RLQ pain.

There are several limitations to this study. First, our retrospective method of analysis required us to develop a system of definitions for a positive and negative scan result. The sonographers and radiologists who reported the scans used diverse terminology in describing the findings, ranging from definitive statements to hedging. Second, we assumed that patients who clinically improved, were discharged from hospital and did not re‐present did not harbour appendicitis. This is presumably true for the vast majority, although grumbling or resolving appendicitis is a clinical possibility.

Further education of referrers and ultrasound users combined with continuous improvements in ultrasound imaging may further improve the diagnostic performance of ultrasound in the detection of appendicitis. Future studies may be useful to monitor this progress. Furthermore, we urge other centres to compare their diagnostic performance in the detection of acute appendicitis because of the high variability of sensitivity and specificity reported in the literature.

## 5 Conclusion

The sensitivity of ultrasound in the detection of appendicitis had a prevalence of 16% in our predominantly young female cohort, with alternative gynaecological and abdominal diagnoses identified approximately 2:1. The sensitivity for ultrasound to detect appendicitis was 60%, with a specificity of 98% and NPV of 93%. In the absence of any ultrasound features, appendicitis is unlikely. These results support the continued use of ultrasound as the primary imaging modality for the assessment of patients with right lower quadrant pain.

## Ethics Statement

Ethical approval for this study was granted by the nominated local institutional ethics committee (Health New Zealand/Te Whatu Ora Waikato Clinical Audit Support Unit) with study reference number 4304P. Individual patient consent was not required, as the study involved retrospective analysis of existing imaging and report data.

## Conflicts of Interest

The authors declare no conflicts of interest.

## Data Availability

The data that support the findings of this study are available from the corresponding author upon reasonable request.
